# The performance of metagenomic next-generation sequencing in diagnosing pulmonary infectious diseases using authentic clinical specimens: The Illumina platform versus the Beijing Genomics Institute platform

**DOI:** 10.3389/fphar.2023.1164633

**Published:** 2023-04-17

**Authors:** Shuangyu Han, Zhan Zhao, Lei Yang, Jie Huang, Yubao Wang, Jing Feng

**Affiliations:** Department of Respiratory and Critical Care Medicine, Tianjin Medical University General Hospital, Tianjin, China

**Keywords:** metagenomic next-generation sequencing, Illumina, BGI, conventional examination, pulmonary infection

## Abstract

**Introduction:** Metagenomic next-generation sequencing (mNGS) has been increasingly used to detect infectious organisms and is rapidly moving from research to clinical laboratories. Presently, mNGS platforms mainly include those from Illumina and the Beijing Genomics Institute (BGI). Previous studies have reported that various sequencing platforms have similar sensitivity in detecting the reference panel that mimics clinical specimens. However, whether the Illumina and BGI platforms provide the same diagnostic performance using authentic clinical samples remains unclear.

**Methods:** In this prospective study, we compared the performance of the Illumina and BGI platforms in detecting pulmonary pathogens. Forty-six patients with suspected pulmonary infection were enrolled in the final analysis. All patients received bronchoscopy, and the specimens collected were sent for mNGS on the two different sequencing platforms.

**Results:** The diagnostic sensitivity of the Illumina and BGI platforms was notably higher than that of conventional examination (76.9% vs. 38.5%, *p* < 0.001; 82.1% vs. 38.5%, *p* < 0.001; respectively). The sensitivity and specificity for pulmonary infection diagnosis were not significantly different between the Illumina and BGI platforms. Furthermore, the pathogenic detection rate of the two platforms were not significantly different.

**Conclusion:** The Illumina and BGI platforms exhibited similar diagnostic performance for pulmonary infectious diseases using clinical specimens, and both are superior to conventional examinations.

## 1 Introduction

Pulmonary infections cause significant morbidity and mortality annually worldwide, leading to various complications such as empyema, pleural effusion, and lung abscess (Magill et al., 2014). Pulmonary infections are immune-mediated lung diseases caused by various microbial pathogens, including fungi, bacteria, viruses, atypical pathogens, and parasites. Early recognition and verification of the pathogen and treatment with appropriate antibiotics are critical to improving outcomes in pulmonary infections. Conversely, delays could lead to disease worsening and a greater risk of death. For an extended period, detection of pathogenic bacteria has mainly relied on conventional examination (CE), such as smears, culture, immunological tests, and polymerase chain reaction (PCR).

Sputum samples, fiber bronchoscope brush biopsies, bronchoalveolar lavage fluids (BALF), and endobronchial biopsies are the most common respiratory specimen types. However, one problem in detecting pathogens is that conventional pathogen detection methods are time-consuming because an infectious disease may be caused by a wide range of pathogens, which must be checked individually. Another limitation is that antibiotic treatment significantly reduces the diagnostic efficacy in the culture, and the pathogens in some infectious diseases cannot be detected. Moreover, given the notable drawbacks of CE, treatment decisions are largely more empirical, particularly the emergence of mixed infection and multidrug-resistant bacteria, making further treatment difficult. Therefore, a novel pathogen detection method for improved detection and precise treatment is urgently required.

Metagenomic next-generation sequencing (mNGS) technology has been used to identify the etiology of infection and potential pathogens, including viruses, parasites, bacteria, and fungi, using high-throughput sequencing without the need to isolate and cultivate individual isolates. In the fields of clinical microbiology, compared with CE methods, mNGS has shown significant advantages, including unbiased detection, high throughput sequencing, and relatively rapid turnaround; it takes only approximately 24 h on a basic NGS workflow, which includes sample/library preparation, sequencing, data analysis, and reporting. Consequently, the mNGS technology shows evident advantages in clinical utility with its rapid identification of pathogens and simultaneous detection of multiple pathogens. It is widely used to complement CE methods and has been increasingly applied in clinical and public health settings.

NGS technology has increasingly developed, and different sequencing platforms have been applied for mNGS of clinical samples. Among the numerous available sequencing platforms, second generation sequencing technologies, such as the platforms provided by Illumina and the Beijing Genomics Institute (BGI), are the most commonly used (Jerome et al., 2019; Zhou et al., 2019; Chen L et al., 2020; Chen P et al., 2020; Yan et al., 2020; Liu et al., 2021; Zhao et al., 2021). However, few studies have determined whether choosing different sequencing platforms significantly affects clinical diagnosis; hence, selecting an appropriate sequencing platform remains a challenge for clinical laboratories and clinicians. Previous studies have reported that various sequencing platforms have similar sensitivity in detecting reference panels that mimic clinical specimens (Liu et al., 2021). However, the reference samples may not precisely represent clinical specimens. To address this issue, we investigated the diagnostic accuracy of the BGI and Illumina platforms by detecting clinical specimens simultaneously to aid clinicians in interpreting the diagnostic results of different platforms, which helps to guide treatment decisions. We attempted to evaluate the advantages of mNGS for detecting pathogens and the difference between the Illumina and BGI platforms in terms of their diagnostic performance for pulmonary infectious diseases.

## 2 Materials and methods

### 2.1 Study design and patient population

**FIGURE 1:**
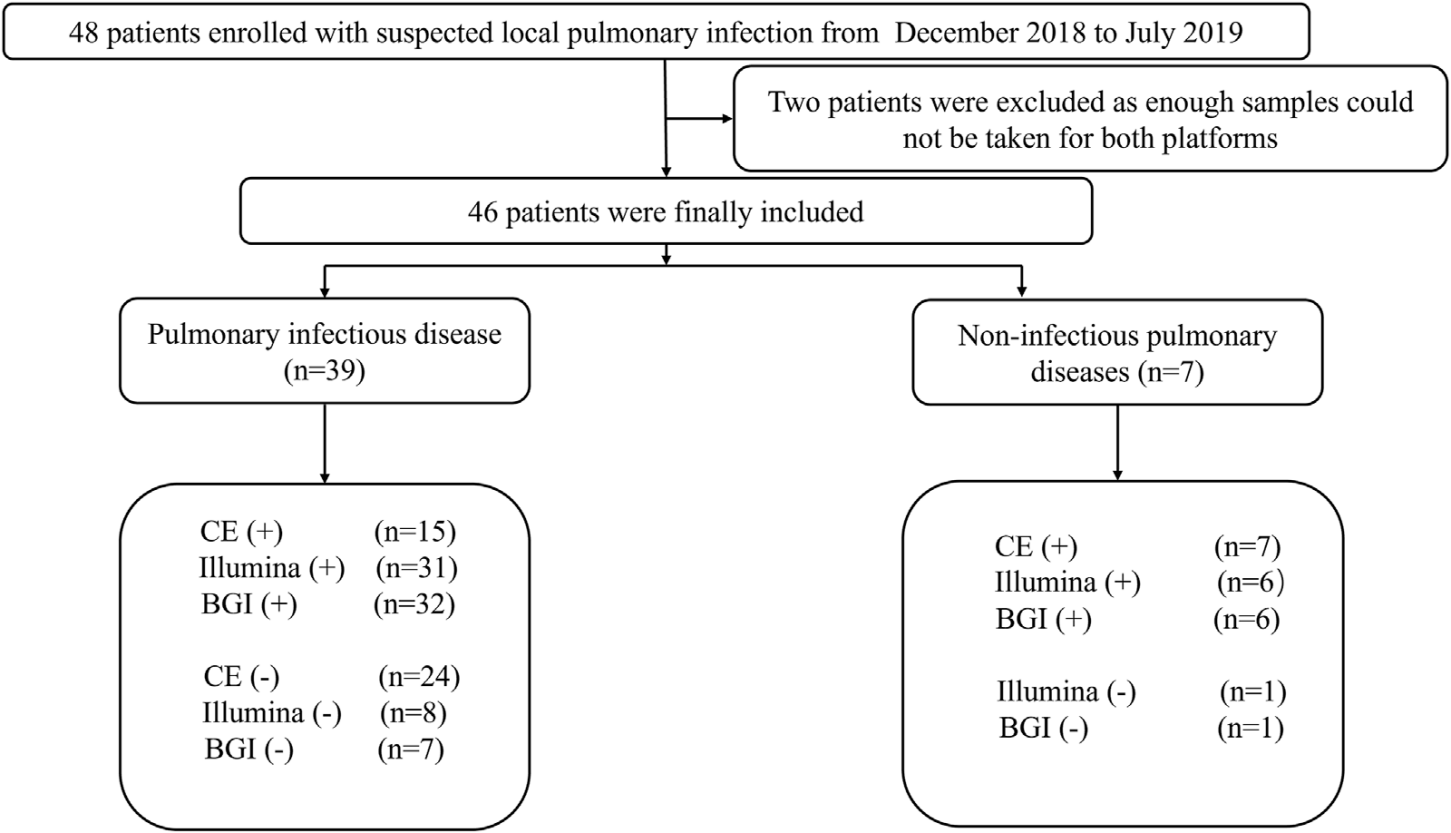
Flow chart depicting the enrollment of patients in this study. CE, conventional examination; BGI, Beijing Genomics Institute.

This prospective, observational, single-center study was conducted in the Respiratory Department of the General Hospital of Tianjin Medical University, China, from December 2018 to July 2019. The major inclusion criteria were as follows: 1) patients with suspected pulmonary infection who exhibited typical clinical symptoms of pulmonary infection, such as cough, fever, expectoration, and respiratory failure, and laboratory data and radiographic manifestations of pneumonia; 2) patients that agreed to undergo bronchoscopy and mNGS testing; 3) both specimens and the detection process passed mNGS quality control; and 4) patients with complete medical records. Patients with incomplete medical records and no mNGS results were excluded. A total of 46 patients admitted to the hospital with suspected pneumonia were analyzed (Figure 1).

### 2.2 Specimen collection and processing

The specimens collected included transbronchial lung biopsy (6–10 pieces, each weighing approximately 4–6 g) and BALF. Furthermore, fiber bronchoscope brush biopsies of patients with suspected pulmonary infection were collected using bronchoscopy performed according to standard procedures (Meyer et al., 2012).

Samples from three different categories were collected from most patients; only one or two samples were collected in some patients because of long-term bronchoscopy intolerance or difficulty in obtaining specimens. Subsequently, the lung biopsy specimens were sent to histopathology laboratories within 2 h, and dehydration, paraffin embedding, slicing, special pathological staining (including hematoxylin-eosin (HE) staining, Ziehl–Neelsen acid-fast staining, hexamine silver staining), and pathological examination were carried out. The BALF samples were used for fungal and bacterial culture, galactomannan (GM) test, and GeneXpert MTB/RIF (Xpert) assays for diagnosing tuberculosis (TB). The remaining lung biopsy specimens, BALF, and brush biopsies were divided into two parts and sent to the BGI and Illumina for mNGS. Other CEs, including sputum culture, serum GM test, β-1,3-glucans test (G-test), and cryptococcal capsular antigen serology detection, were also performed. For cytomegalovirus (CMV) and Ebstein-Barr virus (EBV) detection in blood, quantitative real-time polymerase chain reaction (qRT-PCR) was conducted.

### 2.3 mNGS on two sequencing platforms and data analysis

#### 2.3.1 BGI platform

DNA was extracted from lung biopsy specimens, BALF, and brush biopsy samples using the TIANamp Micro DNA Kit (DP316; TIANGEN BIOTECH, Beijing, China) following the manufacturer’s standard procedures. The Agilent 2100 equipment (Agilent Technologies, Santa Clara, CA, United States) was used for quality control of the DNA libraries. Libraries that passed quality control were sequenced using the BGISEQ-50 platform (Jeon et al., 2014a). To obtain high-quality sequencing data, low-quality and short (length <35 bp) reads were removed. Computational subtraction databases of human host sequences were mapped to the human reference genome (GRCh38 downloaded from NCBI: ftp://ftp.ncbi.nlm.nih.gov/genomes/on December 2013) using the Burrows-Wheeler alignment (BWA) tool (version 0.7.17-r1188, http://biobwa.sourceforge.net/) (Li et al., 2009). Microbial reads were classified using Kraken2 (version 2.1.2). The classification reference databases were downloaded from NCBI (ftp://ftp.ncbi.nlm.nih.gov/genomes/). RefSeq contains more than 4,000 viral genomes, nearly 3,000 bacterial genomes, over 200 clinically prevalent fungi, as well as 140 disease-related parasites.

#### 2.3.2 Illumina platform

DNA from lung samples including lung biopsy specimens, BALF, and brush biopsies was extracted using the TIANamp Micro DNA kit (DP316; Tiangen Biotech, Beijing, China). The extracted DNA was amplified into 200–300 bp fragments, and a DNA library was constructed by terminal overnight repair and PCR amplification of extracted DNA. The libraries were sequenced on the Illumina MiniSeq platform (Illumina) (Gu et al., 2019). Additionally, read quality was determined using FASTQC. High-quality sequencing data were retained, while short (<35 bp), low-complexity, and low-quality reads were removed, followed by computational subtraction of human sequences using the BWA tool. Reads were aligned to the GRCh38 human reference genome using BWA (version. 0.7.17-r1188). Finally, the remaining reads were aligned to the Microbial Genome Database. Microbial reads were classified using Kraken2 (version 2.1.2). The classification reference databases were downloaded from NCBI (ftp://ftp.ncbi.nlm.nih.gov/genomes/). RefSeq contains 4192 whole-genome sequence of viral taxa, 3233 bacterial genomes or scaffolds, 265 fungi related to human infection, and 274 parasites associated with human diseases.

### 2.4 Diagnostic criteria

Owing to the lack of standard methods for interpreting mNGS results and the diversity of reported parameters among different sequencing platforms, we used previously published standards (Petrucelli et al., 2020; Xie et al., 2021). The interpretation criteria were as follows: 1) a relative abundance of bacteria and fungi at the genus level (excluding *Mycobacterium tuberculosis*) greater than 30%; 2) sample considered positive for *M. tuberculosis* when at least one read aligned to the reference genome at the genus or species level, and 3) a pathogen considered to be positively detected using a traditional detection method and the mNGS reads number more than 50 (Li et al., 2018). An extensive literature search showed that for identifying pathogens of pulmonary infection, the normal skin flora and microbes in the oral cavity or respiratory tract must be excluded. The final clinical mNGS diagnosis results were determined by combining the original mNGS results and the clinical symptoms of the patients, which were analyzed thoroughly by three experienced physicians. CE pathogenic diagnosis was performed if at least one of the following criteria was satisfied: 1) positive culture results of the sputum, lung tissue, BALF, or transbronchial needle aspiration; 2) positive GeneXpert TB result of lung tissue sample DNA, sputum, or BALF; 3) presence of pathogens or granulomas related to infections, detected with Ziehl–Neelsen, HE, Grocott’s methenamine silver, and periodic acid-Schiff staining for examination of tissue pathology; or 4) CMV/EBV DNA levels >1,000 copies/mL in the blood detected using qRT-PCR were considered CMV/EBV positive.

Immunosuppression status was defined as the concurrent use of an immunosuppressive agent for hematologic or solid malignancy, ongoing severe cytopenia, solid organ transplantation, or hematopoietic stem cell transplantation (Zachariah et al., 2020).

### 2.5 Statistical analysis

Statistical analysis was performed using SPSS 28.0. Count data are expressed as the percentage of the number of cases (n%). Associations between groups were determined using two-by-two contingency tables. Sensitivity (Se), specificity (Sp), negative predictive value (NPV), and positive predictive value (PPV) are presented with their 95% confidence intervals (CIs). Comparisons were performed using the McNemar test and Cochran’s Q test. *p* < 0.05 was considered significant.

## 3 Results

### 3.1 Baseline characteristics of patients

In total, 46 patients with suspected pulmonary infection were enrolled in the final analysis (Figure 1). Their baseline characteristics are shown in Table 1. Twenty (43.48%) patients were female, and 26 (56.52%) were male. The mean age was 40.96 ± 19.40 years. Thirty-three (71.73%) patients had an immunosuppressed status. Furthermore, all patients underwent bronchoscopy. The main samples obtained during bronchoscopy were BALF (42 cases, 91.30%), bronchoscopy lung biopsy tissue (34 cases, 73.91%), protected specimen brush (34 cases, 73.91%), and lymph node biopsy (2 cases, 4.34%). Amongst the 46 patients, 39 (84.78%) were diagnosed with pulmonary infection, and seven (15.22%) showed no clinical evidence of pulmonary infection (non-infectious pulmonary disease, Figure 1).

**TABLE 1:** Baseline characteristics of the patients (N = 46).

	Total patients’ group [n (%)]
**Sex**	
Male	26 (56.52)
Female	20 (43.48)
**Age (years) (mean ± SD)**	40.96 ± 19.40
<40	22 (47.83)
40–70	22 (47.83)
≥70	2 (4.34)
**Sample type (mNGS)**	
BALF	42 (91.30)
Lung tissue	34 (73.91)
Brush	34 (73.91)
**Chest computed tomography (CT)**	
Bilateral	26 (56.52)
Unilateral	20 (43.48)
**Immunocompromised status**	
Immunocompromised	33 (71.73)
Acute Lymphocytic Leukemia	14 (30.43)
Acute Myeloid Leukemia	10 (21.74)
Myelodysplastic Syndrome	2 (4.35)
Multiple Myeloma	2 (4.35)
Aplastic Anemia	2 (4.35)
Non-Hodgkin’s lymphoma	1 (2.17)
Systemic Lupus Erythematosus	1 (2.17)
Idiopathic Thrombocytopenia	1 (2.17)
Non-immunocompromised	13 (28.26)

**FIGURE 2:**
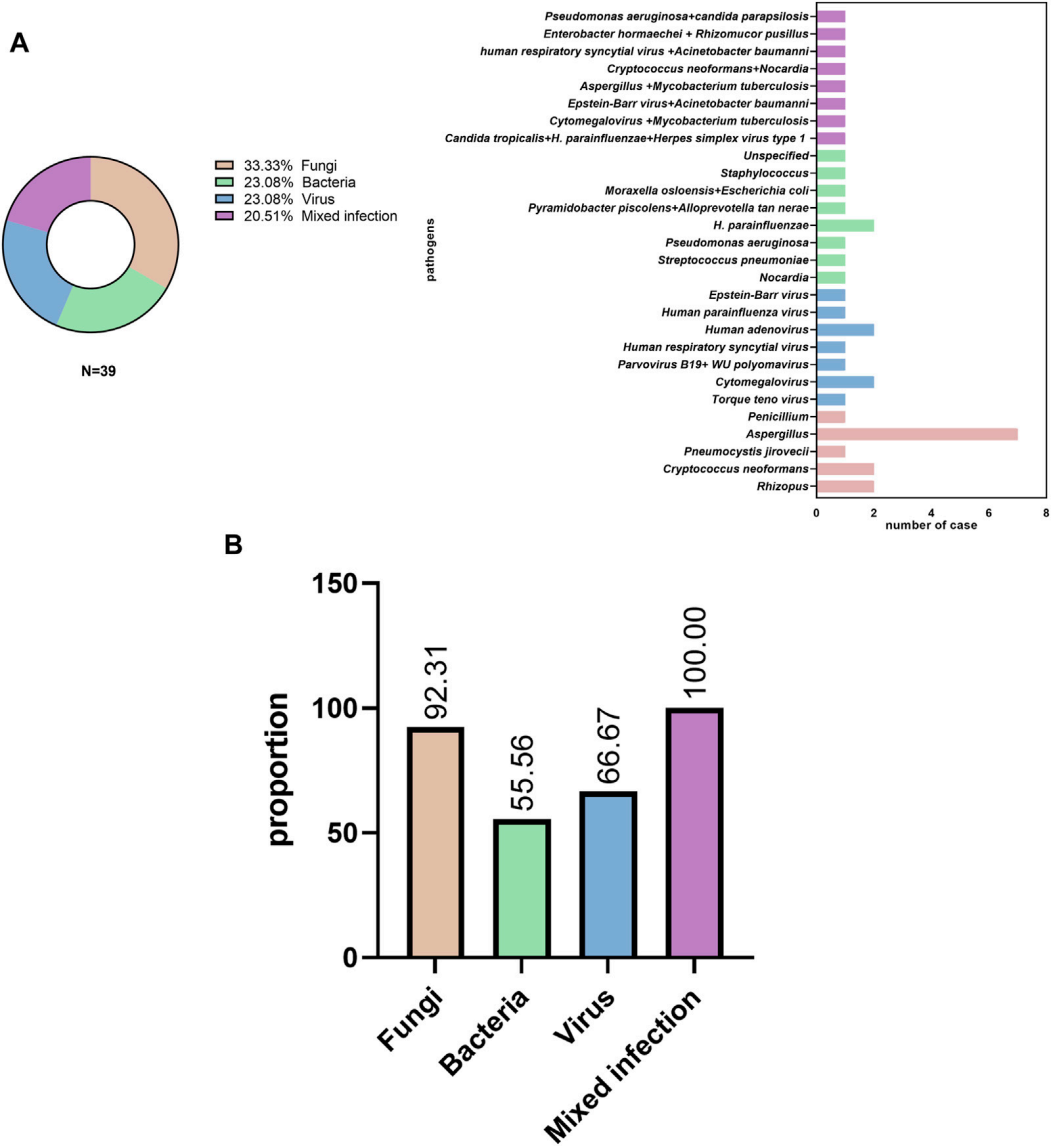
Pathogen distribution **(A)** in pulmonary infectious disease and **(B)** initial empiric antibiotic treatment.

A number of patients were infected with the following pathogens: 1) fungi (13 cases, 33.3%), including *Aspergillus*, *Rhizopus*, *Cryptococcus*, and *Pneumocystis jirovecii*; 2) bacteria (9 cases, 23.08%), including *Streptococcus pneumoniae*, *Haemophilus influenzae*, *Pseudomonas aeruginosa*, *Nocardiopsis*, *Moraxella osloensis*, *Escherichia coli*, and *Staphylococcus*; 3) viruses (9 cases, 23.08%), including Epstein-Barr virus*, human* respiratory syncytial virus*, human parvovirus B19*, *Torque teno virus*, *human adenovirus*, and *human parainfluenza virus*; and 4) mixed infection (8 cases, 20.51%), including co-infection with *Aspergillus* and *M. tuberculosis*, *Cryptococcus* and *Nocardia*; Enterobacteriaceae, *K. pneumoniae*, *Mycoplasma pneumoniae*, and *Mucor circinelloides*; and Cytomegalovirus and *M. tuberculosis* (Figure 2A).

Most patients (31/39, 79.49%) received empirical antibiotic therapy during hospitalization. Among patients with fungal, bacterial, viral, and mixed infections, antibiotics were used in 12 of 13 cases (92.31%), 5 of 9 cases (55.56%), 6 of 9 cases (66.67%), and 8 of 8 cases (100%), respectively (Figure 2B).

### 3.2 Comparison of CE and mNGS (Illumina vs. BGI) in the diagnosis of pulmonary infection

**FIGURE 3:**
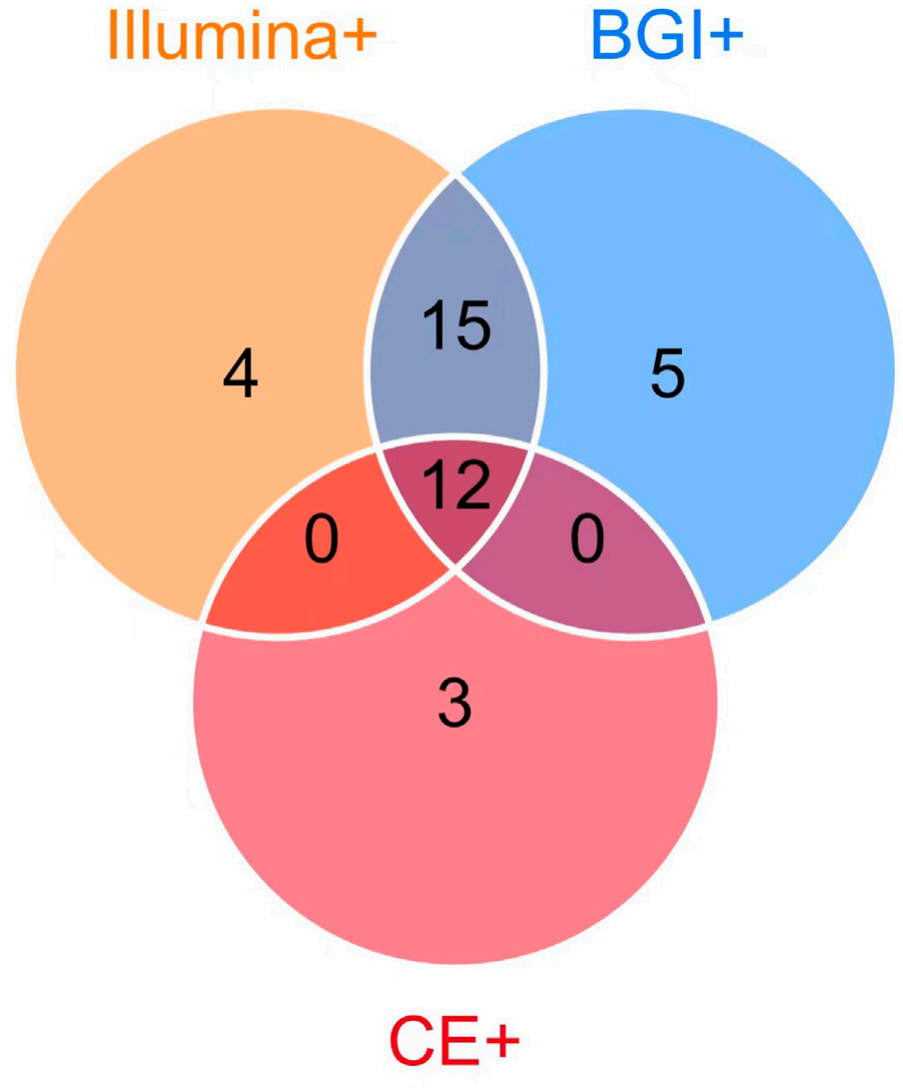
Comparison of pathogens detected using mNGS (Illumina and BGI platforms) and CE. Among the 39 patients with identified pathogens, 15 (38.46%) were diagnosed using CE (pink circle) and 3 (7.69%) using CE only; 36 (92.31%) were diagnosed using mNGS, 32 (82.05%) using the BGI platform (blue circle), 31 (79.49%) using the Illumina platform (orange circle), 12 (30.77%) using mNGS and CE (the overlap of pink, orange, blue circle). The 27 patients (69.23%) were diagnosed using the Illumina and BGI platforms (overlap between orange and blue circle Illumina and BGI platforms), 4 (10.25%) using only the Illumina platform, and 5 (12.82%) using only the BGI platform.

Among the 39 patients with identified pathogens, 15 (38.46%) cases were diagnosed using CE, 31 (79.49%) using only the Illumina platform, and 32 (82.05%) using only the BGI platform (Figure 1). Twenty-seven (69.23%) cases were diagnosed using both the Illumina and BGI platforms, 4 (10.25%) cases using only the Illumina platform, and 5 (12.82%) cases using only the BGI platform (Figure 3). Furthermore, three (7.69%) cases were not detected by both platforms (Figure 3). Among the seven patients with non-infectious pulmonary disease, six (85.71%) cases were diagnosed using both the Illumina and BGI platforms, and seven (100%) cases were diagnosed using CE (Figure 1).

The performance of the two mNGS platforms and CE in diagnosing suspected pulmonary infection is shown in Table 2. The Se, Sp, PPV, and NPV of the Illumina platform were 76.9% (95% CI: 64.46–89.23), 85.7% (95% CI: 48.71–97.42), 96.8% (95% CI: 88.43–99.46), and 40% (95% CI: 21.38–67.40), respectively. Meanwhile, those of the BGI platform were 82.1% (95% CI: 67.33–91.02), 85.7% (95% CI: 48.71–97.42), 97.0% (95% CI: 84.68–99.47), and 46.2% (95% CI: 23.22–70.84), respectively. Furthermore, those of CE were 38.5% (95% CI: 24.89–54.10), 100% (95% CI: 64.58–100.0), 100% (95% CI: 79.62–100), and 22.6% (95% CI: 11.39–39.81), respectively. The Se of the Illumina and BGI platforms were significantly higher than that of the CE (76.9% vs. 38.5%, *p* < 0.05; 82.1% vs. 38.5%, *p* < 0.05); however, the Se of the three methods did not have significant difference (100% vs. 85.7%, *p* > 0.05). In addition, the Se and Sp of the two platforms showed no significant difference (76.9% vs. 82.1%, *p* > 0.05; 85.7% vs. 85.7%, *p* > 0.05).

**TABLE 2:** Comparison of the sensitivity and specificity between the mNGS platforms (BGI and Illumina platform) and conventional examinations.

Method	% sensitivity	% specificity	% PPV	% NPV
CE	38.5 (24.89–54.10)	100 (64.58–100.0)	100 (79.62–100)	22.6 (11.39–39.81)
Illumina	76.9^a^ (64.46–89.23)	85.7^a^ (48.71–97.42)	96.8^b^ (88.43–99.46)	40^b^ (21.38–67.40)
BGI	82.1^a^ ^,^ ^c^ (67.33–91.02)	85.7^a^ ^,^ ^c^ (48.71–97.42)	97.0^c^ (84.68–99.47)	46.2^c^ (23.22–70.84)

Data are presented as percent and 95% confidence interval. PPV, positive predictive value; NPV, negative predictive value; mNGS, metagenomic next-generation sequencing; CE, conventional examination. *p* < 0.05 was considered statistically significant.

^a^ Compared to CE, *p* < 0.001.

^b^ Compared to CE, *p* > 0.05.

^c^ Compared to Illumina, *p* > 0.05.

### 3.3 Comparison of the diagnostic performance between CE and the mNGS platforms (Illumina vs. BGI)

**FIGURE 4:**
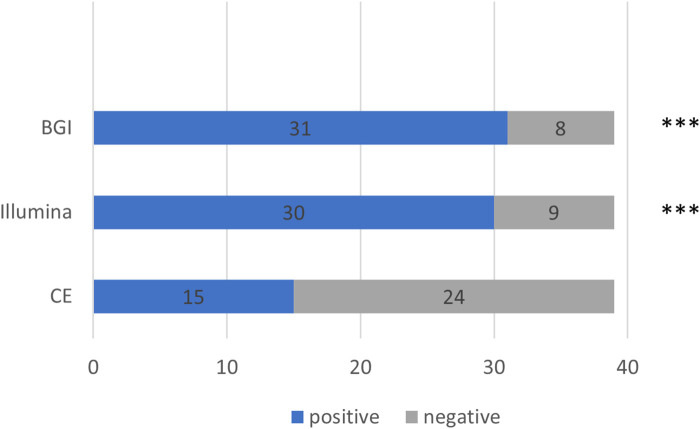
Comparison between the pathogenic detection rates of CE and the mNGS platforms (Illumina and BGI). ***p < 0.001, compared with CE. The *x*-axis represents the number of cases, while the *y*-axis represents the detection methods. CE, conventional examinations.

We further compared the detection rates of the Illumina and BGI platforms and CE in the 39 patients with pulmonary infections (Cochran Q = 20.222, *p* < 0.001) (Figure 4). The detection rate of CE was significantly inferior to that of the Illumina and BGI platforms (*p* < 0.001). No significant difference was observed between the detection rates of the Illumina and BGI platforms (*p* > 0.05) (Figure 4).

**FIGURE 5:**
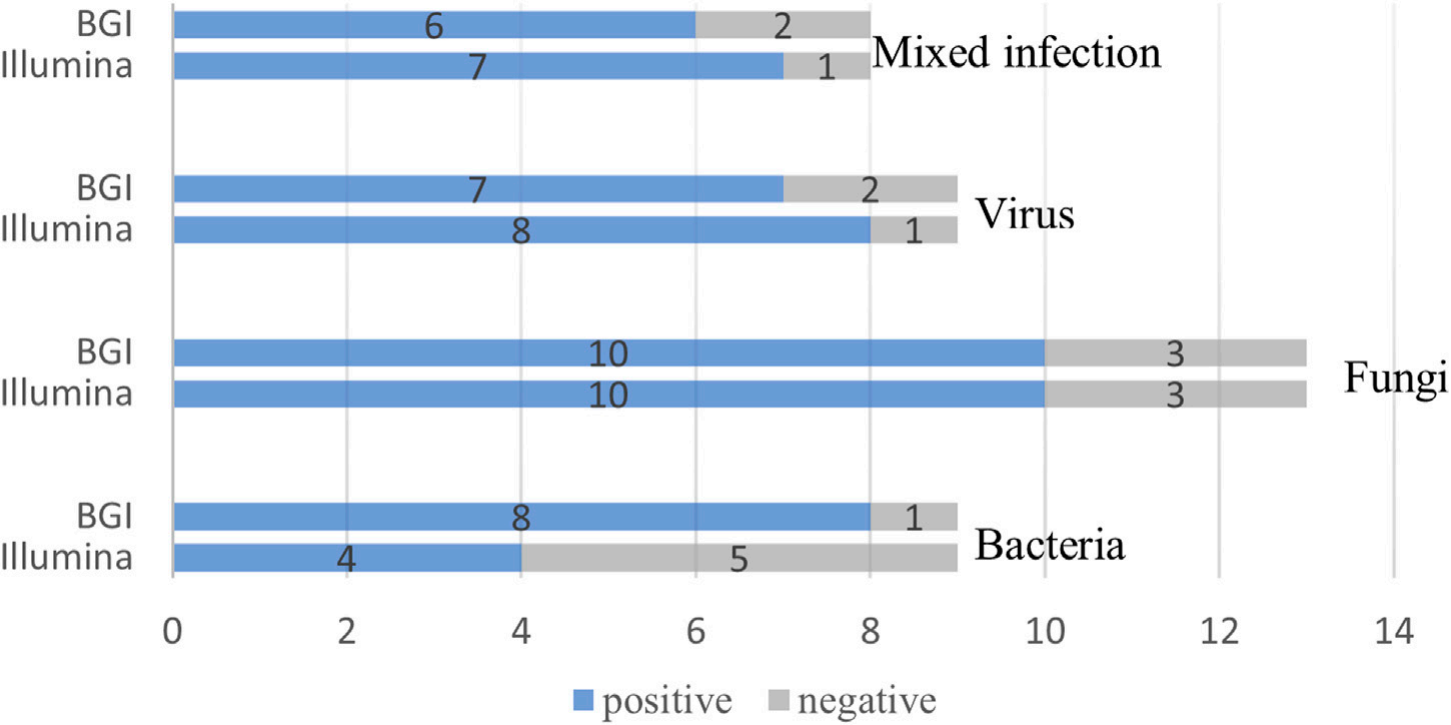
Comparison of the Illumina and BGI platforms in detecting different pathogens. No significant difference was observed between the two platforms (*p* > 0.05). The number of positive samples (*x*-axis) for pairwise Illumina and BGI platforms is plotted against the bacteria, fungi, virus and mixed infection groups (*y*-axis).

We compared the diagnostic performance of the Illumina and BGI platforms in detecting bacterial, fungal, viral, and mixed infections. Of the nine pulmonary bacterial infection cases, five were detected using Illumina and eight using BGI (*p* = 0.39). Of the 13 fungal infections, 10 were detected using Illumina and 10 using BGI (*p* > 0.99). Eight of the nine pulmonary virus infections were detected using Illumina, while seven were detected using BGI (*p* > 0.99). Lastly, seven of the eight mixed infections were detected using Illumina, and six were detected using BGI (*p* > 0.99) (Figure 5).

## 4 Discussion

In the present study, the most commonly detected pathogens were bacteria, viruses, fungi, and mixed infections. The Se of the Illumina and BGI platforms was significantly higher than that of the CE. A previous study found that mNGS had higher Se and Sp than CE (Cai et al., 2020). However, our findings were consistent with those of other published studies (Li et al., 2018; Wang et al., 2019; Huang et al., 2020). These discrepancies may have been caused by the low sample size of our study.

Furthermore, we found that both the Illumina and BGI platforms had a high detection rate of pulmonary infection compared to CE. These results are consistent with those of a previous study (Miao et al., 2018; Zhao et al., 2021). In the present study, nine patients were diagnosed with bacterial infection (Figure 2A); amongst them, only 22.22% (2/9) were identified using CE. One representative case was diagnosed with lymph node bacterial infection by pathology of massive neutrophil infiltration. Another case was diagnosed with *P. aeruginosa* infection detected using BALF culture. However, these findings differ substantially from those of a prior report in which compared with mNGS, the traditional culture method identified the vast majority (74%) of bacterium-associated pneumonia (Toma et al., 2014). We consider that the lower positive culture rate in this study may be related to the initial empiric antibiotic treatment (Figure 2B).

Furthermore, in this study, the Illumina and BGI platforms offer numerous advantages in detecting *Aspergillus* infection, which is consistent with a previous study (Gu et al., 2021; Yang et al., 2021). We found that both the Illumina and BGI platforms detected *Aspergillus* infection in 100% (7/7) of patients diagnosed, and only 28.57% (2/7) of such cases were detected using a serological GM test (not a significant difference). Moreover, a previous study identified immunocompromised status as a risk factor associated with fungal and bacterial co-infection (Zhao et al., 2021). Among the eight cases of mixed pulmonary infection confirmed in the present study, eight (100%) had immunosuppressed status, seven (87.50%) mixed infections were diagnosed with the Illumina platform, and six (87.50%) were diagnosed as mixed infections using the BGI platform (*p* > 0.05). Unsurprisingly, some pathogens were not detected using CE in the eight mixed infections.

It is worth mentioning that two cases were diagnosed with TB. A patient (P12) was diagnosed with co-infection with *Aspergillus* and *M. tuberculosis*, and another patient (P30) was diagnosed with co-infection with Cytomegalovirus and *M. tuberculosis*. *M. tuberculosis* was detected using the TB GENE XPERT test of BALF and mNGS. The result is consistent with that of previous studies (Zhou et al., 2019; Xu et al., 2022), which showed that two platforms had a similar detection ability for *M. tuberculosis* when compared with TB GENE XPERT. However, because of the small sample sizes, these studies had limited power to detect effects; therefore, more evidence is needed.

CE is recommended as an auxiliary or necessary detection method for microorganisms. Three cases (P01, P03, and P41) diagnosed with cryptococcal infection, *Penicillium* infection, and bacterial lymphadenitis were detected using CE in our study. The pathogen causing pulmonary infection was unknown in one patient (P41) who was diagnosed based on histopathological examination, which showed an abundant infiltration of neutrophils.

The mNGS can theoretically report all pathogens with known genome sequences. This also means that microbial contamination in the human body will confuse doctors or affect clinical decisions (Pragman et al., 2018). Owing to the lack of standardization, defining a “positive” or “negative” infection can be difficult, thereby complicating the process of interpretation and comparisons of mNGS results. Moreover, the cost of mNGS is relatively high, which may also affect its clinical application.

Illumina sequencers are characterized by sequencing-by-synthesis technology, which includes solid-phase amplification and a cyclic reversible termination process, which confers high coverage, generating very deep data and short reads with high accuracy. However, the major shortcomings of Illumina are the long run times and the low throughput (Jerome et al., 2019; Chen L et al., 2020). Meanwhile, the BGISEQ-50 platform is based on sequencing-by-ligation technology, which consists of combinatorial probe-anchor synthesis and DNA nanoballs. It has a short sequencing time, high output, and low cost; however, short read lengths are produced (Zhou et al., 2019; Chen P et al., 2020; Yan et al., 2020). In our study, we explored whether the samples from patients sequenced using different platforms yield the same diagnosis, which commonly confuses physicians. We found no difference in the pathogenic detection rate of the Illumina and BGI platforms (Figure 5). Thus, we can choose either of the two platforms, which does not significantly affect the clinical diagnosis. This study is helpful to clinicians selecting platforms of mNGS.

Our study has certain limitations. One is the small sample size. However, although experienced senior clinicians made a clinical judgment, no unified criteria are available for detecting pathogens using mNGS. Furthermore, distinguishing between pathogenic and colonizing microorganisms is still a challenge. Moreover, 79.49% of patients were given empirical antimicrobic treatment before pathogen detection, which might decrease the rate of obtaining positive results from the culture. CE methods of virus detection are limited in our laboratory, which may decrease the rate of virus detection to some extent.

In summary, the sensitivity of both the Illumina and BGI platforms in pulmonary infection pathogen detection were notably higher than that of CE. Furthermore, with the combination of CE and clinician experience, this technology will become a promising tool for diagnosing infectious diseases and developing a tailored therapy. Moreover, the Illumina and BGI platforms had similar performances in the diagnosis of pulmonary infectious diseases. Thus, for clinicians, the choice of sequencing platform has a less influence on clinical diagnosis. However, considerable work still needs to be done in interpreting the mNGS results.

## Data Availability

The data presented in the study are deposited in the NCBI. The accession number is PRJNA942244.
